# Serum Uric Acid Level and Intraventricular Hemorrhage in Patients with Acute Hemorrhagic Stroke: A Retrospective Observational Study

**DOI:** 10.34172/aim.31804

**Published:** 2025-01-01

**Authors:** Su Jin Choi, Min Wook So, Sunggun Lee, Seung Won Choi, Doo-Ho Lim

**Affiliations:** ^1^Department of Internal Medicine, Ulsan University Hospital, University of Ulsan College of Medicine, Ulsan, South Korea; ^2^Department of Internal Medicine, Pusan National University Yangsan Hospital, Pusan National University School of Medicine, Yangsan, South Korea; ^3^Department of Internal Medicine, Haeundae Paik Hospital, Inje University College of Medicine, Busan, South Korea

**Keywords:** Intracerebral hemorrhage, Intraventricular hemorrhage, Uric acid

## Abstract

In contrast to experimental studies indicating the neuroprotective role of uric acid (UA), recent clinical studies have shown that increased UA is associated with a risk of acute hemorrhagic stroke. However, the association of UA with intraventricular hemorrhage (IVH) has not been adequately evaluated. In this study, we determined the relationship between UA and IVH in patients with intracerebral hemorrhage (ICH). We included 721 patients with ICH who were admitted to a tertiary hospital in South Korea. The patients were stratified into quartiles based on their UA levels. IVH decreased continuously across all quartiles of UA. After adjusting for confounding factors, the odds ratio (OR) for IVH was significantly lower in the fourth quartile compared with that in the first quartile (OR: 0.713; 95% CI: 0.546–0.934; *P*=0.045). In conclusion, UA is independently associated with IVH, suggesting its protective role against IVH in patients with ICH.

## Introduction

 Intracerebral hemorrhage (ICH) is a life-threatening type of stroke accounting for 10~15% of all stroke.^1^ Intraventricular hemorrhage (IVH) occurs in approximately 45% of spontaneous ICH cases, and patients with IVH are twice more likely to have a poor clinical outcome and almost three times more likely to die compared with patients without IVH.^2^ In contrast to experimental study conducted in animal models that suggest a neuroprotective role for uric acid (UA),^3^ recent clinical studies in humans have demonstrated that elevated UA levels are associated with increased risk of ICH.^4,5^ However, the association of UA with IVH has seldom been studied. Therefore, in this study, we aimed to investigate the serum UA levels in patients with spontaneous ICH and assessed its relationship with IVH.

## Materials and Methods

###  Study Population and Data Collection

 This retrospective study was conducted on 1059 ICH cases who were admitted through the emergency room to a referral hospital in South Korea, between August 2008 and December 2017. Among these patients, we excluded (1) ICH patients caused by any other vascular conditions such as aneurysmal rupture, moyamoya disease, arteriovenous malformation, coagulopathy, or traumatic ICH (n = 247), and (2) patients who did not undergo laboratory study of UA at the time of visit (n = 2). Patients who visited the emergency room several times for recurrent ICH (n = 87) were included only for the first visit. Finally, out of these 1059 cases, 721 were chosen to be included in the study.

 The baseline demographic data, including age, sex, smoking status, and comorbidity conditions, were obtained from an electrical medical record database. Hypertension was defined as a documented history of systolic blood pressure ≥ 140 mm Hg, diastolic blood pressure ≥ 90 mm Hg, or the use of antihypertensive medications. Hyperlipidemia was defined as a total cholesterol level ≥ 240 mg/dL, low-density lipoprotein cholesterol ≥ 160 mg/dL, or the use of lipid-lowering medications. Smoking status was classified into three categories: never smokers, former smokers, and current smokers, based on patient self-reports. Diabetes mellitus was defined as a documented history of the condition or the use of anti-diabetic medications. Clinical and laboratory data, such as body mass index; blood pressure; Glasgow Coma Scale; and serum UA, serum cholesterol, and C-reactive protein levels, were obtained for each patient at the time of their visit to the emergency room.

 Computed tomographic (CT) angiography was performed as the primary radiological diagnostic method. IVH was defined as the presence of blood in the ventricular system detected on initial brain CT angiography performed at the time of admission. We did not have a long-term follow-up period for the development of delayed IVH after ICH. The volume of ICH was measured using brain CT by multiplying the horizontal and vertical dimensions of the hemorrhage observed in each slice, providing an estimate of the hemorrhage volume by summing the product of these dimensions across all affected slices.^6^ The location of ICH was categorized into two main regions: supra-tentorial ICH, which includes hemorrhages located in the cerebral hemispheres, and infra-tentorial ICH, which encompasses hemorrhages in the cerebellum and brain stem.^2^ The data collected were based on the initial CT scan conducted upon the patient’s arrival at the hospital, and IVH presence was recorded as an acute complication.

###  Statistical Analysis

 The participants were stratified into quartiles (viz., quartile 1, ≤ 3.90 mg/dL; quartile 2, 3.91–5.00 mg/dL; quartile 3, 5.01–6.10 mg/dL; quartile 4, ≥ 6.11 mg/dL) according to their serum UA levels. Categorical variables were expressed as frequencies with percentages and continuous variables as the mean and standard deviation. Between-group comparisons were performed using Pearson’s chi-square test for categorical variables, and one-way analysis of variance was used for numerical variables, as appropriate. Multivariate analysis was performed using a logistic regression model to analyze the association between serum UA levels and the presence of IVH. Covariates in the multivariable analysis, which were selected according to their clinical importance and statistical significance, included age, sex, systolic and diastolic blood pressure, smoking status, triglyceride, C-reactive protein levels, the volume of ICH and the location of ICH. All *P* values were two-sided, and *P* < 0.05 was considered statistically significant. Data manipulation and statistical analyses were performed using the SPSS software (Version 24; SPSS Inc., Chicago, IL, USA).

## Results

 Of 721 patients with ICH, 413 (57.3%) were male. The mean age of the patients was 58.9 ± 14.03 years. The mean serum UA level was 5.94 ± 1.70 mg/dL. The baseline characteristics of the study population according to the quartiles for serum UA are listed in Table 1. The prevalence of male sex, systolic and diastolic blood pressure, and serum triglyceride and C-reactive protein levels increased significantly with serum UA quartile. The proportion of never-smokers and the presence of IVH were lower with increasing serum UA quartiles.

**Table 1:** Baseline Characteristics of the Study Population According to the Quartiles of Serum Uric Acid Levels

**Characteristics **	**Overall** **(** ***n*** **=721)**	**Serum uric acid**				
		**Quartile 1** **≤3.90 mg/dL** **(** ***n*** **=184)**	**Quartile 2** **3.91 – 5.00 mg/dL** **(** ***n*** **=182)**	**Quartile 3** **5.01 – 6.10 mg/dL** **(** ***n*** **=172)**	**Quartile 4** **≥6.11 mg/dL** **(** ***n*** **=183)**	***P *** **Value**
Age, years	58.9 ± 14.0	63.3 ± 14.7	59.0 ± 14.0	58.8 ± 12.9	54.6 ± 13.2	0.085
Male gender	413 (57.3)	60 (32.6)	82 (45.1)	120 (69.8)	151 (82.5)	< 0.001
Body mass index, kg/m^2^	23.9 ± 4.8	22.6 ± 3.6	24.3 ± 7.9	23.9 ± 4.0	24.7 ± 3.7	0.657
Systolic blood pressure, mm Hg	172.5 ± 34.5	164.8 ± 31.0	170.9 ± 30.4	174.2 ± 35.3	180.0 ± 39.0	0.002
Diastolic blood pressure, mm Hg	98.6 ± 22.3	92.3 ± 19.0	98.5 ± 21.3	101.4 ± 22.3	102.2 ± 25.1	0.027
Initial Glasgow coma scale	11.6 ± 4.2	11.5 ± 4.2	11.7 ± 4.2	11.4 ± 4.4	11.8 ± 4.0	0.150
Never smoker	393 (54.5)	127 (69.0)	117 (64.3)	83 (36.1)	66 (36.1)	< 0.001
Hypertension	432 (60.3)	103 (56.9)	104 (57.1)	105 (61.8)	120 (60.3)	0.272
Diabetes mellitus	129 (18.0)	36 (19.9)	33 (18.1)	31 (18.2)	29 (15.8)	0.796
Hyperlipidemia	38 (5.3)	12 (6.6)	11 (6.0)	6 (3.5)	9 (4.9)	0.585
Previous stroke	80 (11.2)	21 (11.6)	20 (11.0)	22 (12.9)	17 (9.3)	0.747
Coronary artery disease	6 (3.3)	7 (3.8)	11 (6.5)	9 (4.9)	35 (2.8)	0.510
White blood cell, /mL	10,389.0 ± 5,346.0	10,482.6 ± 4,419.1	9,573.8 ± 3958.0	10,798.7 ± 7,645.9	10,718.6 ± 4,698.9	0.092
Hemoglobin, g/dL	13.820 ± 1.9301	13.116 ± 1.7911	13.518 ± 1.7817	14.227 ± 1.6989	14.448 ± 2.1195	0202
Platelet, /mL	230,396.4 ± 81,840.6	240,255.4 ± 87,970.5	222,348.1 ± 75,330.3	233,175.4 ± 76,532.3	225,847.0 ± 85,837.7	0.501
Total cholesterol, mg/dL	186.7 ± 44.6	181.0 ± 41.9	189.9 ± 43.5	192.0 ± 43.6	184.1 ± 48.6	0.409
LDL cholesterol, mg/dL	98.4 ± 37.9	77.1 ± 27.9	96.5 ± 35.9	120.9 ± 35.1	97.0 ± 42.0	0.821
HDL cholesterol, mg/dL	50.3 ± 14.8	56.3 ± 16.1	56.8 ± 16.7	42.7 ± 11.2	46.5 ± 11.0	0.440
Triglyceride, mg/dL	112.7 ± 79.0	60.4 ± 18.0	98.3 ± 105.6	136.7 ± 63.7	150.5 ± 79.5	0.003
GFR, mL/min/1.73m^2^	80.3 ± 23.8	84.7 ± 24.0	83.8 ± 21.5	81.46 ± 23.0	71.3 ± 24.3	0.379
C-reactive protein, mg/L	0.57 ± 2.1	0.53 ± 1.1	0.40 ± 1.0	0.55 ± 1.6	0.81 ± 3.4	0.013
Volume of ICH, cm^3^	19.5 ± 25.7	20.1 ± 25.9	19.1 ± 26.7	19.4 ± 23.8	19.5 ± 25.9	0.107
Supra-tentorial ICH	616 (85.4)	157 (85.3)	161 (88.5)	147 (85.5)	151 (82.5)	0.458
Presence of IVH	276 (38.3)	85 (46.2)	68 (37.4)	64 (37.2)	59 (32.2)	0.049

Values are shown as mean ± standard deviation or number (%). LDL, Low-density lipoprotein; HDL, High-density lipoprotein cholesterol; GFR, Glomerular filtration rate; ICH, intracerebral hemorrhage; IVH, Intraventricular hemorrhage.

 The association between serum UA and the presence of IVH is listed in Table 2. Using the first quartile as the reference value, the odds ratio (OR) of IVH decreased as the serum UA quartile increased; however, it was signiﬁcantly lower in the fourth serum UA quartile (OR: 0.554; 95% confidence interval [CI]: 0.363–0.847; *P* = 0.006). Furthermore, the adjusted odds ratio for IVH remained statistically signiﬁcant in the fourth serum UA quartile (OR: 0.713; 95% CI: 0.546–0.934; *P* = 0.045) even after adjustment for covariates, including age, sex, systolic and diastolic blood pressure, smoking status, triglyceride, C-reactive protein levels, the volume of ICH and the location of ICH.

**Table 2:** Association between Serum Uric Acid Level and Intraventricular Hemorrhage According to the Quartiles of Serum Uric Acid

**Intraventricular Hemorrhage**	**Univariable**		**Multivariable**	
	**Odds Ratio (95% CI)**	***P *** **Value**	**Odds Ratio (95% CI)**	***P *** **Value**
Quartile 1 (reference)	1		1	
Quartile 2	0.803 (0.518 – 1.244)	0.326	0.987 (0.650 – 1.502)	0.790
Quartile 3	0.727 (0.473 – 1.117)	0.145	0.929 (0.746 – 1.152)	0.619
Quartile 4	0.554 (0.363 – 0.847)	0.006	0.713 (0.546 –0.934)	0.045

CI, confidence interval Covariates in the multivariable model include age, sex, systolic blood pressure, diastolic blood pressure, smoking status, triglyceride level, and C-reactive protein levels.

## Discussion

 In this study, we found that serum UA level was an independent predictor of IVH after adjusting for cerebrovascular risk factors. The occurrence of IVH was lower with increasing serum UA quartiles, suggesting a protective role against IVH in ICH patients.

 The role of UA in hemorrhagic stroke has not been defined. Several studies have suggested the detrimental effects of UA during hemorrhagic stroke. For example, the pro-oxidant effect of UA may cause vascular smooth cell proliferation and endothelial dysfunction, resulting in inadequate control of blood pressure and the progression of arterial stiffness.^7,8^ In contrast, other studies have shown that UA has a neuroprotective effect because of its antioxidant role in scavenging free oxygen radicals. Moreover, UA may decrease cerebral amyloid angiopathy, which is considered an important pathologic factor in ICH.^9,10^

 IVH is a devastating complication associated with ICH that can lead to worsening morbidity and mortality.^2^ Although there have been several studies describing a relationship between UA and ICH,^7,9^ there are few reports on the relationship between UA and IVH. While some reports suggested that elevated serum UA levels might correlate with favorable outcomes of IVH, findings remain inconsistent across different populations and clinical settings, indicating that the relationship may be influenced by various underlying factors, including age, the severity of hemorrhage, and comorbid conditions.^2,11,12^ In this study, our findings indicate that elevated serum UA is associated with the lower presence of IVH. Whether UA *per se* exerts a protective effect against the development of IVH or is only a marker during protection remains unclear. Therefore, large-scale randomized studies or experimental studies should be conducted to elucidate the role and mechanism of UA in the progression of IVH.

 Our study had several limitations. First, it was a retrospective, single-center, and existing data study, which introduces potential for selection bias and confounding effects. Nevertheless, this design enabled us to analyze a relatively large sample of real-world clinical data, incorporating diverse patient characteristics and information that may not be feasible in randomized clinical trials. To address these limitations, we used comprehensive statistical adjustments, including multivariable regression, to mitigate biases. Therefore, despite these inherent limitations, we believe that our study provides meaningful insights that enhance understanding of the relationship between serum UA and IVH. Second, we lacked data on socioeconomic status and detailed information regarding the duration and treatment of hypertension, which may influence ICH outcomes and could limit the generalizability of our results. Third, our volume and location measurements for ICH were based on standard imaging methods; however, variations in imaging modality or timing could introduce minor measurement inconsistencies. Finally, the patients in this study consisted of only one ethnic group (Korean), so the applicability of our results to other ethnic groups may be limited.

 In conclusion, in this cross-sectional study, high serum UA level was associated with a low risk of IVH in patients with ICH, suggesting its neuroprotective effect. Further large-scale, prospective studies are warranted to clarify the specific role of UA in IVH progression.
